# Dietary requirements of synthesizable amino acids by animals: a paradigm shift in protein nutrition

**DOI:** 10.1186/2049-1891-5-34

**Published:** 2014-06-14

**Authors:** Guoyao Wu

**Affiliations:** 1 Department of Animal Science, Texas A&M University, College Station, Texas 77843, USA

**Keywords:** Diet, Metabolism, Nutrition, Protein, Requirements

## Abstract

Amino acids are building blocks for proteins in all animals. Based on growth or nitrogen balance, amino acids were traditionally classified as nutritionally essential or nonessential for mammals, birds and fish. It was assumed that all the “nutritionally nonessential amino acids (NEAA)” were synthesized sufficiently in the body to meet the needs for maximal growth and optimal health. However, careful analysis of the scientific literature reveals that over the past century there has not been compelling experimental evidence to support this assumption. NEAA (e.g., glutamine, glutamate, proline, glycine and arginine) play important roles in regulating gene expression, cell signaling, antioxidative responses, fertility, neurotransmission, and immunity. Additionally, glutamate, glutamine and aspartate are major metabolic fuels for the small intestine to maintain its digestive function and to protect the integrity of the intestinal mucosa. Thus, diets for animals must contain all NEAA to optimize their survival, growth, development, reproduction, and health. Furthermore, NEAA should be taken into consideration in revising the “ideal protein” concept that is currently used to formulate swine and poultry diets. Adequate provision of all amino acids (including NEAA) in diets enhances the efficiency of animal production. In this regard, amino acids should not be classified as nutritionally essential or nonessential in animal or human nutrition. The new Texas A&M University’s optimal ratios of dietary amino acids for swine and chickens are expected to beneficially reduce dietary protein content and improve the efficiency of their nutrient utilization, growth, and production performance.

## Introduction

Amino acids (AA) are building blocks for proteins and must be present in cells for synthesis of polypeptides [1]. The carbon skeletons of eleven of these AA (namely cysteine, histidine, isoleucine, leucine, lysine, methionine, phenylalanine, threonine, tryptophan, tyrosine, and valine) are not synthesized from non-AA molecules in cells of any animals [2]. Therefore, they are classified as nutritionally essential AA (EAA) and must be included in diets for nonruminants to maintain physiological functions of cells, tissues, and the whole body [3,4]. This assumes particular importance for the small intestine because its basal membrane lacks an ability to take up a nutritionally significant quantity of all AA, except for glutamine, from the arterial circulation [5,6].

Classical animal nutrition textbooks do not consider cysteine or tyrosine as an EAA [7-10], because they can be synthesized from methionine and phenylalanine in the liver, respectively. However, the inability of all animals to form the carbon skeletons for methionine and phenylalanine means that there is no *de novo* synthesis of cysteine or tyrosine [2]. Also, intestinal mucosal cells must depend on cysteine and tyrosine as essential precursors to synthesize polypeptides [6]. Moreover, sulfur-containing or aromatic AA in arterial blood are largely not available to enterocytes (absorptive columnar cells in the small intestine). Thus, the presence of cysteine and tyrosine in diets, which can reduce the dietary needs of their precursor AA, are *necessary* to maintain the normal structure and function of the intestine [5,6].

Rose did consider dietary needs of some of the traditionally classified NEAA in his human studies in the 1940s and 1950s, and reported that the omission of NEAA from the diet did not affect nitrogen balance in healthy adults during an eight-day experimental period [8]. Thus, it has long been assumed that all of the “nutritionally nonessential amino acids (NEAA)” are synthesized sufficiently in the body to meet the needs for maximal growth and optimal health [7-10]. However, careful analysis of the scientific literature reveals that over the past century there has not been compelling experimental evidence to support this assumption [2]. Indeed, in the 1960s and 1970s, A.E. Harper and other investigators found that the absence of NEAA from chicken and rat diets could not support maximal growth of these animals [11-15]. Growing evidence shows that nearly all of these synthesizable AA are inadequately present in typical plant protein (e.g., corn- and soybean meal)-based diets for growing swine relative to optimal whole-body protein synthesis [16]. Results of recent research revealed that the NEAA have important regulatory roles in nutrient metabolism to favor lean tissue growth and reduction of white adipose tissue [17-20]. Clearly, animals have dietary requirements for not only EAA, but also NEAA to achieve maximum growth and production performance [21-23]. This new concept has resulted in a paradigm shift in our understanding of protein nutrition and is highlighted in the present review article.

### Definitions of requirements of dietary AA

Requirements of dietary AA can be classified as qualitative and quantitative [2]. Qualitative requirements are related to the question of “*what* AA are required for maintenance, optimum performance (e.g., growth, lactation, reproduction, and sports competition), and optimum health (e.g., prevention of chronic metabolic disorders, resistance to infectious disease, and recovery from illness)?” Quantitative requirements refer to the question of “*how much* of an AA is required for maintenance, optimum growth, and optimum health?” Feeding experiments have traditionally been employed to determine both qualitative and quantitative requirements of dietary AA by animals [24]. Minimal requirements of AA can also be estimated using so-called factorial analysis, that is, measurements of the loss of N by animals fed a nitrogen- or AA-free diet via urine, feces, gas, and other routes (maintenance) + AA deposited in animals + AA excreted as animal products (e.g., milk, egg, wool, and fetus) [2]. Over the past three decades, studies involving radioactive and stable AA tracers have been used along with the N balance technique to determine dietary requirements for EAA by humans and farm animals [25,26]. The more modern methods involve the use of direct and indirect indicators of AA oxidation during a period of several hours [26]. For yet unknown reasons, the AA oxidation methods generally yielded much higher values of dietary EAA requirements by humans than the nitrogen-balance studies. Readers are referred to recent articles [2,3,24] for insight into historical developments of dietary AA requirements. At present, little is known about dietary requirements for NEAA by mammals, birds, or fishes.

### Development of the ideal protein concept in animal nutrition

#### Chickens

Beginning in the late 1950s, Mitchell and Scott at the University of Illinois conceptualized an ideal protein (optimal proportions and amounts of EAA) for diets of chickens [27,28]. NEAA were not considered by these authors. Early attempts to define an ideal protein were based on the EAA composition of eggs and casein, but were largely unsuccessful because of the excess of many EAA. In 1960, Scott’s group simulated the profile of EAA in the chick carcass [29] to design a revised pattern of dietary EAA in an ideal protein for improving growth performance of chicks [30]. An improvement in the ideal protein was indeed achieved using this approach, but remained unsatisfactory due to the lack of NEAA in the diet. However, data on the composition of all EAA or NEAA in chicks were not available [29]. Subsequently, a mixture of several AA (cystine, glycine, proline and glutamate), which are synthesized from pre-existing AA (including EAA) by birds and had previously been thought to be NEAA in chicken nutrition, was used in dietary formulations to yield better results on growth performance [31,32]. This extensive research during the 1960s and the 1970s culminated in several versions of the “chick AA requirement standard” for the first three weeks post-hatching [33-36]. Reference values were given in the Dean and Scott Standard [33], the Huston and Scott Reference Standard [34], the modified Sasse and Baker Reference Standard [35], and the Baker and Han’s Ideal Chick Protein [36] (Table 1). The common features shared by these different recommended standards of dietary AA requirements by chickens are that the diets included: (a) all EAA that are not synthesized by chickens; (b) several AA (cystine, glutamate, glycine, proline, and tyrosine) that are synthesized from either EAA or α-ketoglutarate plus ammonia by animals to various extents; and (c) no data on alanine, aspartate, asparagine, glutamine, or serine. Note that the patterns of AA composition in the ideal protein for chicks, as proposed by the Scott [33,34] and Baker [25,36], differ substantially for glycine and proline, and, to a lesser extent, for branched-chain AA, histidine, and sulfur-containing AA. These differences may reflect variations in AA composition of chickens reported in the literature. Because the content of proline plus hydroxyproline in the body of chickens was not known at that time, the relatively small amount of proline in the recommended ideal protein was only arbitrarily set and could limit responses of the animals to dietary EAA in their maximal growth and production performance. In contrast, very large amounts of glutamate (e.g., 13 times the lysine value in the modified Sasse and Baker Reference Standard) [35] were used to presumably provide for the entire need for “nonspecific AA N”. However, key questions regarding whether glutamate fulfilled this role and whether excess glutamate might interfere with the transport, metabolism and utilization of other AA in chickens were not addressed by the Illinois investigators [33-36]. Possibly due to these concerns and the publication of the NRC nutrient requirements for poultry in 1994 [37], Baker [38] did not include glutamate, glycine or proline in an ideal protein for diets of 0- to 56-d-old chickens in his modified University of Illinois Ideal Ratios of Amino Acids for broiler chickens in 1997 (Table 2).

**Table 1:** **The University of Illinois patterns of amino acid compositions in ideal proteins for chicks during the first three wk post-hatching**^**1**^

**Amino acid**	**Amino acid content in the carcass Ref.**[29]**(% of crude protein)**^**2**^	**Dean & Scott standard (1965) Ref.**[33]		**Huston & Scott standard (1968) Ref.**[34]		**Sasse & Baker standard (1973) Ref.**[35]		**Baker & Han standard (1994) Ref.**[36]	
		**Amount in diet**^**3**^	**% of lysine**	**Amount in diet**^**3**^	**% of lysine**	**Amount in diet**^**3**^	**% of lysine**	**Amount in diet**^**3**^	**% of lysine**
Arginine	6.65	1.10	98.2	1.00	105	0.95	104	0.95	106
Cystine	---	0.35	31.3	0.35	36.8	0.35	38.5	0.325	36.1
Glycine	---	1.60	143	1.20	126	0.60	65.9	0.60	66.7
Isoleucine	4.35	0.80	71.4	0.60	63.2	0.60	65.9	0.60	66.7
Histidine	1.80	0.30	26.8	0.30	31.6	0.33	36.3	0.32	35.6
Leucine	7.2	1.20	107	1.20	126	1.00	110	0.98	109
Lysine	6.6	1.12	100	0.95	100	0.91	100	0.90	100
Methionine	1.98^†^	0.55	49.1	0.35	36.8	0.35	38.5	0.325	36.1
Phenylalanine	4.25	0.68	60.7	0.50	52.6	0.50	55.0	0.50	55.6
Proline	---	1.00	89.3	0.20	21.1	0.40	44.0	0.40	44.4
Threonine	4.4	0.65	58.0	0.65	68.4	0.65	71.4	0.60	66.7
Tryptophan	0.98	0.23	20.5	0.15	15.8	0.15	16.5	0.145	16.1
Tyrosine	---	0.63	56.3	0.45	47.4	0.45	49.5	0.45	50.0
Valine	5.0	0.82	73.2	0.82	86.3	0.69	75.8	0.69	76.7
Glutamic acid^4^	---	12.0	1071	10.0	1053	12.0	1319	12.0	1333
Total amino acids		23.0		18.7		19.9		19.8	
Total nitrogen		2.83		2.33		2.37		2.35	

^1^These ideal protein models were developed for 0- to 21-d-old broilers using crystalline amino acids. It was assumed that all of these amino acids were 100% available for absorption into enterocytes in chicks. Except for glycine and methionine, all amino acids are L-isomers. DL-methionine is used herein.

^2^Average values for 1-wk-old and 4- to 5-wk-old chicks.

^3^% of diet (as-fed basis; 90% dry matter).

^4^Provided as the nitrogenous source for synthesis of NEAA in chicks.

^†^This value refers to L-methionine.

**Table 2:** The modified Baker and NRC patterns of changes in amino acid compositions in ideal proteins for 0- to 56-d-old chicks (% of lysine in diet)

**Amino acid**	**Baker’s modified models (1997) for chicks**[38]^**1**^			**NRC (1994)**[37]**(0- to 21-d-old chicks)**^**5**^
	**0 to 21 d**^**2**^	**21 to 42 d**^**3**^	**42 to 56 d**^**4**^	
Lysine	100	100	100	100
Methionine	36	37	37	42
Cystine	36	38	38	33
Threonine	67	70	70	67
Valine	77	80	80	75
Arginine	105	108	108	104
Tryptophan	16	17	17	17
Isoleucine	67	69	69	67
Leucine	109	109	109	100
Histidine	35	35	35	29
Phe + Tyr	105	105	105	112

^1^These ratios are based on true digestible levels of amino acids in diet (as-fed basis; 90% dry matter). Adapted from Baker [38]. Except for glycine, all amino acids are L-isomers.

^2^Patterns of amino acid composition in the ideal protein are the same for male and female chickens. The amounts of digestible lysine in diet (as-fed basis; 90% dry matter) are 1.12% and 1.02% for male and female chickens, respectively.

^3^Patterns of amino acid composition in the ideal protein are the same for male and female chickens. The amounts of digestible lysine in diet (as-fed basis; 90% dry matter) are 0.89% and 0.84% for male and female chickens, respectively.

^4^Patterns of amino acid composition in the ideal protein are the same for male and female chickens. The amounts of digestible lysine in diet (as-fed basis; 90% dry matter) are 0.76% and 0.73% for male and female chickens, respectively.

^5^These ratios are based on total amino acids in a typical corn- and soybean meal-based diet. The amount of digestible lysine in diet (as-fed basis; 90% dry matter) is 1.2% for 0- to 21-d-old chicks.

#### Swine

Work on the ideal protein for poultry diets laid a foundation for subsequent studies with growing pigs. Thus, the British nutritionist Cole suggested in 1980 that swine diets could be formulated to contain ideal ratios of EAA (with lysine as the reference AA) based on their concentrations in the pig carcass (almost exclusively tissue proteins) [39]. This idea was adopted first by the British Agricultural Research Council (ARC) in 1981 [40] and then by the U.S. National Research Council (NRC) in 1988 [41]. Unfortunately, histidine, arginine, and all synthesizable AA were not included in the ARC’s concept of ideal protein (Table 3). Also, its conceptual foundation based solely on the EAA composition of the body was flawed, because the pattern of AA in the diet does not reflect the composition of AA in the animal [16,42]. This mismatch can be explained as follows: (a) individual AA in the diet undergo extensive catabolism and transformations at different rates in the small intestine; (b) the concentrations of AA in the circulation differ markedly from the relative abundance of AA in the diet; (c) individual AA in plasma have different metabolic fates in different animal tissues; and (d) the abundance of AA in tissue proteins differs greatly from that in the diet [2,16,43]. These major shortcomings limit the usefulness of the early versions of the ideal protein in formulating swine diets for maximal growth or production performance of pigs.

**Table 3:** **Previously proposed amino acid compositions for ideal proteins for 10–20 kg growing pigs**^**1 **^**(% of lysine)**

**Amino acid**	**Amino acid content in the carcass**^**2**^	**ARC (1981) Ref.**[39]^**3**^	**Wang-Fuller (1989) Ref.**[44]^**4,a**^	**Chung-Baker (1992) Ref.**[45]^**4,b**^	**NRC (1998) Ref.**[40]^**5,b**^	**Baker (2000) Ref.**[46]^**4,b**^
Arginine	91	---	---	42	42	42
Glycine	---	---	---	100	---	---
Histidine	47	33	---	32	32	32
Isoleucine	53	55	60	60	54	60
Leucine	111	100	110	100	102	100
Lysine	100	100	100	100	100	100
Met + Cys	49	50	63	60^c^	57^e^	60^c^
Phe + Tyr	100	96	120	95^d^	94^f^	95^d^
Proline	---	---	---	33	---	---
Tryptophan	12	15	18	18	19	17
Threonine	61	60	72	65	62	65
Valine	72	70	75	68	68	68
Glutamate^6^	---	---	826	878	---	---

^1^These ratios are based on true digestible levels of amino acids in diets [40,44-46], except for ARC (1981) [36]. Except for glycine, all amino acids are L-isomers.

^2^Taken from Baker (1997) [38]. The body proteins in 20–45 kg pigs contain 63 g lysine/16 g nitrogen [38].

^3^These ratios are based on total amino acids in the diet. The total level of lysine in the diet is 1.10% (as-fed basis; 90% dry matter).

^4^The diet contains 1.20% true digestible lysine (as-fed basis; 90% dry matter).

^5^The diet contains 1.01% true digestible lysine (as-fed basis; 90% dry matter).

^6^Provided as the nitrogenous source for synthesis of other NEAA in animals.

^a^Dietary requirements are for 25–50 kg gilts.

^b^Dietary requirements are for 10–20 kg pigs.

^c^The ratio of L-methionine to L-cystine is 1:1.

^d^The ratio of L-phenylalanine to L-tyrosine is 53:47.

^e^The ratio of L-methionine to L-cystine is 47:53.

^f^The ratio of L-phenylalanine to L-tyrosine is 64:36.

Dietary AA are required by animals primarily for maintenance (including the synthesis of nonprotein metabolites) and protein accretion [2]. However, the ARC’s ideal protein concept did not take into consideration the relative contribution of maintenance to the total AA needs of the pig [40]. This was due, in part, to technical challenges to accurately determine maintenance requirements of AA, which include replacement of degraded proteins, as well as the use of AA for synthesis of low-molecular-weight substances and ATP production [1]. Between 1989 and 1990, in attempts to improve the original ideal protein concept [39,40], T.C. Wang and M.F. Fuller [45] used gilts in the weight range of 25 to 50 kg to estimate an ideal pattern of dietary AA that included requirements for both maintenance and tissue protein accretion. However, these two authors also failed to consider arginine, histidine or all so-called NEAA in the ideal protein although they used glutamate at 826% of the lysine value to provide nonspecific AA-nitrogen [45]. As for the studies with chickens in the 1960s and 1970s, there were also concerns over the assumptions for inclusion of this high level of glutamate in the swine diet that lacks all other NEAA. While glutamate was used to prepare isonitrogenous diets in the previous studies, none of these investigators considered that animals have a dietary requirement of glutamate for optimal growth and production performance.

Having recognized the need to modify the ideal protein concept for formulating swine diets, D.H. Baker took great efforts between 1990 and 2000 to evaluate dietary requirements of EAA by 10–20 kg swine. In their original study, D.H. Baker and his student T.K. Chung added arginine (42% of lysine), glycine (100% of lysine), histidine (32% of lysine), and proline (33% of lysine) to the basal diet containing 1.2% true digestible lysine and using glutamate at 878% of the lysine value to provide nonspecific AA-nitrogen [46]. However, other synthesizable AA (including alanine, aspartate, asparagine, cysteine, glutamine, serine, and tyrosine) were not considered in the revised version of the ideal protein and the rationale for the use of arginine, glycine, histidine, and proline at different proportions to lysine was not explained [46]. Furthermore, the bases for other assumptions were unknown, including: (a) whether glutamate is an effective precursor for sufficient synthesis of all other AA (including aspartate, glutamine, and serine) in specific tissues (e.g., the small intestine, spleen, and lymph nodes) and the whole body; or (b) whether the high content of glutamate in the diet may affect the transport, metabolism and utilization of other AA in the diet. Furthermore, little attention was paid to inter-organ fluxes of amino acids relative to their intracellular metabolism. For example, only ~5% of dietary glutamate enters the portal circulation in growing pigs [5,6]. In addition, although intracellular glutamate is used to synthesize aspartate, many extra-intestinal tissues and cells (e.g., liver and red blood cells) have a limited ability to take up glutamate from the blood circulation [2]. The 10th edition of the NRC Swine Nutrient Requirements published in 1998 [41] did not recognize the needs of pigs for dietary proline or glycine; therefore, Baker [47] omitted glycine and proline from the last version of his “ideal protein” for swine diet formulations in 2000, as he did in 1997 [38]. Over the past two decades, there have been successful attempts to refine the patterns of some AA in diets for lactating, suckling, weanling, finishing, and gestating pigs by addition of arginine [48-53], glutamine [54-59], glutamate [60-64], proline [65-67], or glycine [68,69], or by determining mammary gland growth, changes of whole-body AA composition, and milk yields in lactating sows [70,71]. The outcomes are increases in neonatal and postweaning growth, lactation performance, and litter size in pigs.

Growing evidence shows that both EAA and NEAA (e.g. arginine, glutamine, glutamate, glycine, and proline) play important roles in regulating gene expression, cell signaling, nutrient transport and metabolism, intestinal microbiota, anti-oxidative responses, and immune responses [1,2]. Based on these lines of compelling evidence from animal studies, Wu and colleagues proposed the new concept of functional AA, which are defined as those AA that participate in and regulate key metabolic pathways to improve health, survival, growth, development, lactation, and reproduction of the organisms [1,2,16]. Metabolic pathways include: (a) intracellular protein turnover (synthesis and degradation) and associated events; (b) AA synthesis and catabolism; (c) generation of small peptides, nitrogenous metabolites, and sulfur-containing substances (e.g., H_2_S); (d) urea cycle and uric acid synthesis; (e) lipid and glucose metabolism; (f) one-carbon-unit metabolism and DNA synthesis; and (g) cellular redox signaling. Functional AA can be nutritionally “essential”, “nonessential”, or conditionally essential AA. Notably, the concept of functional AA in nutrition has also been adopted for fish [72-74], poultry [75-79], and small laboratory animals (e.g., mice and rats) [80-83]. Readers are referred to recent reviews and original research article on these new developments [24,84-101].

### Texas A&M University’s optimal ratios of amino acids in diets for swine and chickens

The carbon skeletons of EAA (including tyrosine and cysteine) are not synthesized from non-AA substances in animals [2]. As noted previously, synthesis of NEAA from EAA in animals is inadequate for their maximal growth, milk production, and reproduction performance or for optimal development and health. Thus, the traditional classification of AA as EAA or NEAA is purely a matter of definition. For example, emerging evidence shows that arginine, glutamine, glutamate, and glycine play important roles in regulating gene expression, cell signaling, antioxidative responses, and immunity [51-56]. Additionally, glutamate, glutamine, and aspartate are major metabolic fuels for enterocytes [6] and also regulate intestinal and neurological development and function [2]. In addition, glutamine is essential for ATP production, synthesis of nucleotides, expression of anti-oxidative genes, and redox signaling in enterocytes [57]. Furthermore, glutamate activates chemical sensing in the gastrointestinal tract and may inhibit degradation of both EAA and NEAA by intestinal microbes [2,60]. Finally, proline and arginine, which are major sources of ornithine for intestinal and placental synthesis of polyamines [42], are essential for DNA and protein synthesis and also participate in protein and DNA methylation, and, thus genetic and epigenetic regulation of cell growth and development [2]. Collectively, animals have dietary requirements for all synthesizable AA to achieve their full genetic potential for growth, development, reproduction, lactation, and resistance to infectious disease [21].

Composition of EAA in feed ingredients and true ileal digestibilities of EAA in swine [41] and poultry [37,90] have been published. As an initial step to define NEAA requirements by animals, we recently determined the composition of all protein-AA in major feedstuffs [86] and in animal tissues [21]. Examples are given in Table 4 for corn grain, soybean meal, sorghum grain, and meat & bone meal. Based on the previous studies of AA biochemistry and nutrition (including AA metabolism and tissue protein gains) in poultry e.g., 3, [37] and swine e.g., [48-69], the author of the present work would like to propose Texas A&M University’s optimal ratios of true digestible AA in diets for swine (Table 5) and chickens (Table 6) during different phases of growth and production. The values for 5- to 10-kg young pigs are based primarily on consideration of: (a) the entry of dietary AA into the portal vein for 30-day-old postweaning pigs, as compared to the accretion of AA in the body [16]; (b) the published data of Baker [47] and NRC [41] on dietary EAA requirements; and (c) the estimated rates of AA synthesis, catabolism and accretion in the body [2,16,88]. Specifically, these estimations are that: (a) rates of *net* synthesis of aspartate, arginine, glutamate, glutamine, glycine, and proline in extra-intestinal tissues of 5–10 kg postweaning pigs are 195, 361, 415, 1149, 331, and 276 mg/kg body weight per day, respectively; (b) rates of catabolism (including oxidation and synthesis of low-molecular-weight substances) of alanine and tyrosine in extra-intestinal tissues are 30% of their rates of accretion in body proteins; (c) rates of catabolism (including oxidation) of leucine and isoleucine in extra-intestinal tissues are 30% and 25% of their rates of accretion in body proteins, respectively; (d) the rate of catabolism (including oxidation) of asparagine in extra-intestinal tissues is 124 mg/kg body weight per day; (e) rates of catabolism (including oxidation) of valine and serine in extra-intestinal tissues are 15% of their rates of accretion in body proteins; (f) the rate of catabolism (including oxidation) of phenylalanine in extra-intestinal tissues is 124 mg/kg body weight per day; and (g) the ratio of phenylalanine to tyrosine is 60:40, whereas the ratio of methionine to cysteine is 1:1. Additionally, the Texas A&M University’s optimal ratios of true digestible AA in diets for gestating and lactating sows are based on previous studies of dietary AA composition [89], ileal true digestibility of AA in feed ingredients (Table 4 and Ref. [41]), composition of EAA and NEAA in the body (Table 4), embryonic/fetal survival and growth [42,49,57], as well as milk production and piglet growth [42,50,57].

**Table 4:** Compositions of amino acids in the whole-body proteins of chicks and pigs

**Amino acid**	**Chicks**^**1**^		**Pigs**^**2**^		**Corn grain**^**3**^	**Soybean meal**^**4**^	**Sorghum grain**^**5**^	**Meat & bone meal**^**6**^
	**mg AA/g protein**	**% of lysine**	**mg AA/g protein**	**% of lysine**	**g AA/100 g foodstuff**			
Alanine	66.3	108	65.7	109	0.71	1.95	0.96	4.78
Arginine	68.5	111	67.7	112	0.38	3.18	0.41	3.67
Asparagine	36.5	59.3	36.0	59.7	0.35	2.10	0.31	2.21
Aspartate	43.1	70.1	42.8	71.0	0.43	3.14	0.36	3.08
Cysteine	15.0	24.4	13.2	21.9	0.20	0.70	0.19	0.49
Glutamine	50.5	82.1	51.2	84.9	1.02	3.80	0.85	2.81
Glutamate	82.9	135	84.6	140	0.64	4.17	1.18	4.05
Glycine	115	187	117	19.4	0.40	2.30	0.39	8.67
Histidine	21.1	34.3	20.8	34.5	0.23	1.13	0.23	1.19
Isoleucine	35.9	58.4	35.3	58.5	0.34	2.03	0.38	1.92
Leucine	69.2	113	68.3	113	1.13	3.44	1.21	3.56
Lysine	61.5	100	60.3	100	0.25	2.80	0.21	3.13
Methionine	18.9	30.7	18.7	31.0	0.21	0.60	0.20	1.10
Phenylalanine	34.8	56.6	34.3	56.9	0.46	2.21	0.51	1.85
Proline	85.3	139	86.1	143	1.06	2.40	0.96	5.86
OH-Proline	34.8	56.6	37.9	62.9	0.00	0.09	0.00	2.88
Serine	45.0	73.2	44.3	73.5	0.45	2.12	0.46	2.08
Threonine	36.3	59.0	35.1	58.2	0.31	1.76	0.32	2.42
Tryptophan	11.6	18.9	11.1	18.4	0.07	0.62	0.10	0.39
Tyrosine	26.6	43.3	27.2	45.1	0.43	1.66	0.45	1.45
Valine	41.8	68.0	42.2	70.0	0.44	2.09	0.50	2.23

Except for glycine, all amino acids are L-isomers. Adapted from Wu et al. [21]. Calculations were based on the molecular weights of intact amino acids. OH-Pro = hydroxyproline.

^1^Chickens (10-d-old). The content of protein in the body is 14.3 g/100 g wet tissue.

^2^Pigs (30-d-old). The content of protein in the body is 14.1 g/100 g wet tissue.

^3^As-fed basis (89.0% dry matter). Corn grain contains 9.3% crude protein (as-fed basis) [91].

^4^As-fed basis (89.0% dry matter). Soybean meal contains 43.6% crude protein (as-fed basis) [91].

^5^As-fed basis (89.1% dry matter). Sorghum grain contains 10.1% crude protein (as-fed basis) [91].

^6^As-fed basis (96.1% dry matter). Meat & Bone meal contains 52.0% crude protein (as-fed basis) [91].

**Table 5:** True ideal digestibilities of amino acids in corn and soybean meals for poultry and swine diets (%)

**Amino acid**	**Chicks**^**1**^				**Pigs**^**2**^			
	**Corn grain**	**Soybean meal**	**Sorghum grain**	**Meat & Bone meal**	**Corn grain**	**Soybean meal**	**Sorghum grain**	**Meat & bone meal**
Alanine	87.6	88.9	85.4	90.2	88.5	89.0	87.2	90.5
Arginine	88.4	90.6	87.2	91.4	89.3	90.2	88.4	91.3
Asparagine	86.5	88.3	85.9	90.6	86.8	88.5	86.0	90.2
Aspartate	87.2	89.5	86.1	90.2	86.3	88.2	85.8	89.7
Cysteine	85.1	86.4	84.8	89.4	86.0	87.1	85.1	89.0
Glutamine	88.6	89.5	87.6	90.8	87.7	89.2	86.8	90.8
Glutamate	89.2	90.2	88.4	91.2	88.1	89.6	87.4	91.0
Glycine	86.4	88.3	85.7	90.5	86.6	88.0	85.7	89.5
Histidine	85.5	87.4	84.9	89.6	87.0	88.5	86.2	90.2
Isoleucine	88.7	89.3	88.0	90.8	88.2	88.9	87.6	90.5
Leucine	88.2	89.0	87.6	90.3	87.8	89.6	86.4	90.6
Lysine	85.0	88.4	84.3	90.0	84.5	89.8	83.7	90.4
Methionine	87.5	90.1	86.8	90.6	88.6	89.1	87.4	90.5
Phenylalanine	89.1	90.3	88.5	90.9	89.5	90.0	88.9	91.0
Proline	86.8	88.0	85.9	89.4	86.4	87.2	86.0	89.2
Hydroxyproline	---	---	---	88.7	---	---	---	88.4
Serine	88.4	90.2	87.5	91.1	88.6	89.0	87.9	90.6
Threonine	85.2	86.5	84.8	89.3	84.9	86.8	84.3	88.5
Tryptophan	86.0	87.2	85.3	89.0	85.2	88.1	84.6	89.7
Tyrosine	88.5	89.6	88.0	91.4	89.0	90.2	88.2	91.0
Valine	88.2	89.8	87.6	90.7	87.1	88.7	86.1	90.3

^1^Except for glycine, all amino acids are L-isomers. Broiler chickens (21-wk-old). Ileal digestae were obtained from 21-d-old broiler chicks [92] at 6 h after they were fed either a nitrogen-free purified diet (consisting of 94.9% cornstarch, 2% soybean oil, 1.65% dicalcium phosphate, 0.5% vitamin premix, 0.5% mineral premix, 0.25% NaCl, and 0.2% chromic oxide) or a diet containing the test feed ingredient (consisting of 79.9% cornstarch, 15% test ingredient, 2% soybean oil, 1.65% dicalcium phosphate, 0.5% vitamin premix, 0.5% mineral premix, 0.25% NaCl, and 0.2% chromic oxide) and then euthanized by cervical dislocation. The amount of the diet was provided to overnight (16 h)-fasted chicks at 25 g/kg body weight. Amino acids in the diet, as well as the digestae from chicks fed either the nitrogen-free purified diet or the diet containing the test feed ingredient were analyzed as described by Li et al. [91,94] to calculate the true ideal digestibilities of AA [2]. Data are means for 8 chicks per ingredient, with pooled SEM values being less than 0.8%.

^2^Pigs (50- to 65-d-old). Ileal digestae were obtained from 50- to 65-d-old cannulated pigs [93] at 6 h after they were fed either a nitrogen-free purified diet (consisting of 94.9% cornstarch, 2% soybean oil, 1.65% dicalcium phosphate, 0.5% vitamin premix, 0.5% mineral premix, 0.25% NaCl, and 0.2% chromic oxide) or a diet containing the test feed ingredient (consisting of 79.9% cornstarch, 15% test ingredient, 2% soybean oil, 1.65% dicalcium phosphate, 0.5% vitamin premix, 0.5% mineral premix, 0.25% NaCl, and 0.2% chromic oxide). The amount of the diet was provided to overnight (16 h)-fasted pigs at 12 g/kg body weight. Amino acids in the diet, as well as the digestae from pigs fed either the nitrogen-free purified diet or the diet containing the test feed ingredient were analyzed as described by Li et al. [91,94] to calculate the true ideal digestibilities of AA [2]. Data are means for 6 pigs per ingredient, with pooled SEM values being less than 1.2%.

**Table 6:** **Texas A&M University’s optimal ratios of true digestible amino acids in diets for swine**^**1 **^**(% of diet (as-fed basis))**

**Amino acid**	**Growing pigs, kg**^**2**^				**Gestating pigs**^**3**^		**Lactating sows**^**2**^
	**5-10**	**10-20**	**20-50**	**50-110**	**d 0-90**	**d 90-114**	
Alanine	1.14	0.97	0.80	0.64	0.69	0.69	0.83
Arginine	1.19	1.01	0.83	0.66	1.03	1.03	1.37
Asparagine	0.80	0.68	0.56	0.45	0.50	0.50	0.66
Aspartate	1.14	0.97	0.80	0.64	0.61	0.61	0.94
Cysteine	0.32	0.28	0.24	0.20	0.19	0.19	0.26
Glutamate	2.00	1.70	1.39	1.12	0.89	0.89	1.81
Glutamine	1.80	1.53	1.25	1.00	1.00	1.60	1.38
Glycine	1.27	1.08	0.89	0.71	0.48	0.48	0.75
Histidine	0.46	0.39	0.32	0.26	0.29	0.29	0.39
Isoleucine	0.78	0.66	0.54	0.43	0.45	0.45	0.66
Leucine	1.57	1.33	1.09	0.87	1.03	1.03	1.41
Lysine	1.19	1.01	0.83	0.66	0.51	0.51	0.80
Methionine	0.32	0.28	0.24	0.20	0.16	0.16	0.25
Phenylalanine	0.86	0.73	0.60	0.48	0.54	0.54	0.77
Proline	1.36	1.16	0.95	0.76	0.89	0.89	1.24
Serine	0.70	0.60	0.49	0.39	0.45	0.45	0.74
Threonine	0.74	0.65	0.55	0.46	0.41	0.41	0.56
Tryptophan	0.22	0.19	0.17	0.14	0.11	0.11	0.18
Tyrosine	0.67	0.57	0.46	0.37	0.40	0.40	0.62
Valine	0.85	0.72	0.59	0.47	0.55	0.55	0.72

^1^Data are taken from Wu [95]. Except for glycine, all amino acids are L-isomers. Values are based on true ileal digestible amino acids. Crystalline amino acids (e.g., feed-grade arginine, glutamate, glutamine and glycine), whose true ileal digestibility is 100%, can be added to a diet to obtain their optimal ratios. The molecular weights of intact amino acids were used for all the calculations. The content of dry matter in all the diets was 90%. The content of metabolizable energy in the diets of growing pigs, gestating pigs, and lactating pigs is 3,330, 3,122, and 3,310 Kcal/kg diet, respectively.

^2^Fed ad libitum (90% dry matter).

^3^Fed 2 kg/d on d 0–90, and 2.3 kg/d on d 90–114 (90% dry matter).

Several additional comments on the Texas A&M University’s optimal ratios of dietary AA are warranted. First, the author adopts the NRC [41] values for lysine, methionine, threonine, and tryptophan in the Texas A&M University’s model for 5- to 10-kg pigs. Second, optimal ratios of EAA in diets of older pigs are based on the suggestions of the NRC [41] and Baker [47] in that the ratios of tryptophan, sulfur-AA, and threonine to lysine (all based on true digestibility of EAA) increase slightly with age, whereas the ratios of other EAA to lysine are not altered substantially during postnatal development. Third, this is the first time that NEAA are included in optimal ratios of dietary AA for pigs and poultry at various physiological stages. Fourth, for EAA, the ratios of BCAA, histidine, phenylalanine or tyrosine to lysine in the Texas A&M University’s optimal ratios of dietary are higher than those proposed by the NRC [41] and Baker [47] for swine. This is based on the following considerations [2]: (a) BCAA are actively degraded in extra-hepatic and extra-intestinal tissues; (b) leucine can stimulate muscle protein synthesis in young pigs; (c) leucine, isoleucine and valine should be in an appropriate ratio to prevent AA imbalance; (d) large amounts of histidine-containing dipeptides are present in skeletal muscle; and (e) tyrosine is actively utilized in multiple metabolic pathways and its carbon skeleton is formed only from phenylalanine in animals. Finally, the data on dietary EAA requirements by chickens [38], along with composition of EAA and NEAA as well as AA accretion in the body (85 and Table 4) and new knowledge of AA metabolism in birds [2], provided the basis for the proposed Texas A&M University’s optimal ratios of amino acids in chicken diets (Table 7). The recommended values for EAA and NEAA requirements must be revised as new and compelling experimental data become available.

**Table 7:** **Texas A&M University’s optimal ratios of true digestible amino acids in diets for chickens**^**1 **^**(% of lysine in diet)**

**Amino acid**	**Age of chickens**		
	**0 to 21 d**^**2**^	**21 to 42 d**^**3**^	**42 to 56 d**^**4**^
Alanine	102	102	102
Arginine	105	108	108
Asparagine	56	56	56
Aspartate	66	66	66
Cysteine	32	33	33
Glutamate	178	178	178
Glutamine	128	128	128
Glycine	176	176	176
Histidine	35	35	35
Isoleucine	67	69	69
Leucine	109	109	109
Lysine	100	100	100
Methionine	40	42	42
Phenylalanine	60	60	60
Proline	184	184	184
Serine	69	69	69
Threonine	67	70	70
Tryptophan	16	17	17
Tyrosine	45	45	45
Valine	77	80	80

^1^Except for glycine, all amino acids are L-isomers. Values are based on true ileal digestible amino acids.

^2^Patterns of amino acid composition in the ideal protein are the same for male and female chickens. The amounts of digestible lysine in diet (as-fed basis; 90% dry matter) are 1.12% and 1.02% for male and female chickens, respectively.

^3^Patterns of amino acid composition in the ideal protein are the same for male and female chickens. The amounts of digestible lysine in diets (as-fed basis; 90% dry matter) are 0.89% and 0.84% for male and female chickens, respectively.

^4^Patterns of amino acid composition in the ideal protein are the same for male and female chickens. The amounts of digestible lysine in diets (as-fed basis; 90% dry matter) are 0.76% and 0.73% for male and female chickens, respectively.

## Conclusion and perspectives

Amino acids have versatile and important physiological functions beyond their roles as the building blocks of protein [101]. Thus, dietary NEAA and EAA are necessary for the survival, growth, development, reproduction and health of animals. Growing evidence shows that pigs and poultry cannot synthesize sufficient amounts of all NEAA to achieve their maximum genetic potential [95-100]. NEAA (e.g., glutamine, glutamate, proline, glycine and arginine) play important roles in regulating gene expression, cell signaling, antioxidative responses, neurotransmission, and immunity. Additionally, glutamate, glutamine and aspartate are major metabolic fuels for the small intestine to maintain its digestive function and to protect its mucosal integrity. While metabolic needs for an AA by animals do not necessarily translate into its dietary needs, results of recent studies indicate that animals have both metabolic and dietary needs for AA that are synthesized in the body [101-115]. Thus, “synthesizable amino acids” should not be considered as “nutritionally nonessential amino acids” in animal feeding. Besides EAA, NEAA and conditionally essential AA should all be taken into consideration in: (a) revising the classical “ideal protein” concept; and (b) formulating balanced diets to improve protein accretion, feed efficiency, and health in animals. The Texas A&M University’s optimal ratios of AA in diets for swine or poultry, as proposed herein, are based on experimental data from published biochemical and nutritional studies. This new initiative will provide a much-needed framework for both qualitative and quantitative analysis of dietary requirements for all AA by livestock, poultry and fish through conduct of additional research. Adoption of these recommended values in animal feeding are expected to beneficially reduce dietary protein content and improve the efficiency of nutrient utilization, growth, and production performance of farm animals. The concept of dietary requirements for NEAA also has important implications in human nutrition and health.

## Competing interests

The author declares no competing interests.

## Authors’ contributions

The author wrote this paper and approved the final version of the manuscript.
